# Improvement of an Effective Protocol for Directed Differentiation of Human Adipose Tissue-Derived Adult Mesenchymal Stem Cells to Corneal Endothelial Cells

**DOI:** 10.3390/ijms222111982

**Published:** 2021-11-05

**Authors:** Cadenas-Martin Marta, Moratilla Adrian, Fernández-Delgado Jorge, Arnalich-Montiel Francisco, Maria P. De Miguel

**Affiliations:** 1 Cell Engineering Laboratory, La Paz University Hospital Health Research Institute, IDiPAZ, 28046 Madrid, Spain; cadenasm95@gmail.com (C.-M.M.); amoratillariofrio@gmail.com (M.A.); 2 Department of Plastic and Reconstructive Surgery, Santa Cristina Hospital and Centrocim, 28009 Madrid, Spain; drfdelgado@gmail.com; 3 Ophthalmology Department, Ramón y Cajal University Hospital, Instituto Ramón y Cajal de Investigación Sanitaria, 28034 Madrid, Spain; francisco.arnalich@salud.madrid.org

**Keywords:** cell reprogramming, differentiation, adipose tissue-derived mesenchymal stem cells, cornea, corneal endothelium

## Abstract

Corneal disease affects 12.5 million individuals worldwide, with 2 million new cases each year. The standard treatment consists of a corneal transplantation from a human donor; however, the worldwide demand significantly exceeds the available supply. Lamellar endothelial keratoplasty, the replacement of only the endothelial layer of the cornea, can partially solve the problem. Progressive efforts have succeeded in expanding hCECs; however, the ability to expand hCECs is still limited, and new sources of CECs are being sought. Crucial advances have been achieved by the directed differentiation of embryonic or induced pluripotent stem cells, but these cells have disadvantages, such as the use of oncogenes, and are still difficult to establish. We aimed to transfer such knowledge to obtain hCECs from adipose tissue-derived adult mesenchymal stem cells (ADSC) by modifying four previously published procedures. We present several protocols capable of the directed differentiation of human ADSCs to hCECs. In our hands, the protocol by Ali et al. was the best adapted to such differentiation in terms of efficiency, time, and financial cost; however, the protocol by Wagoner et al. was the best for CEC marker expression. Our results broaden the type of cells of autologous extraocular origin that could be employed in the clinical setting for corneal endothelial deficiency.

## 1. Introduction

Corneal diseases are a major cause of vision loss, second only to cataracts in overall importance [1]. Approximately 12.5 million individuals worldwide experience unilateral or bilateral vision loss from corneal disease or injury [2], and approximately 2 million new cases are diagnosed each year [3]. Currently, the standard treatment for these conditions consists of a corneal transplantation (keratoplasty) from a human donor, whose success rate is approximately 80%, depending on the patient’s condition, with a <40% transplant survival at 9 years in high-risk scenarios [2]. Moreover, the worldwide demand for donor corneas amply exceeds the available supply. An aging population also decreases the quality of the donations (cornea donors are becoming older) and increases the need for corneas. In fact, there is only one cornea for every seven individuals requiring one in developed countries, and an estimated one for every seventy individuals worldwide [4,5].

Lamellar keratoplasty, i.e., the replacement of only one of the layers of the cornea, can partially solve the problem because it replaces only the diseased or injured layer and contributes to better graft survival. In particular, endothelial keratoplasty (EK) accounts for over one-third of all the corneal grafts performed at present and involves replacing the damaged or pathological corneal endothelium [6,7] using Descemet membrane endothelial keratoplasty (DMEK) or Descemet’s membrane along with a thin stromal tissue for Descemet stripping automated endothelial keratoplasty (DSAEK).

The corneal endothelium is a cell monolayer coating the inner surface of the cornea whose main function is regulating corneal hydration and, thus, its transparency. Human corneal endothelial cells (CEC) are quiescent in vivo and are, therefore, problematic to expand in culture. Progressive efforts by other groups and ours [8,9,10,11,12,13,14] (for a short review, see [15]) have been successful in expanding human CECs. In an experimental animal model, we more recently demonstrated the usefulness of human decellularized stroma as carriers that can improve the visual quality in corneal endothelial disease [16] (for a review on carriers, see [15]). In humans, He Z et al. [17] demonstrated that cultured CECs can be transplanted ex vivo. In a first-in human clinical trial, cultured human CECs supplemented with a rho kinase (Rock) inhibitor were injected into patients with bullous keratopathy. The procedure was safe, and there was no immune response, achieving normal corneal thickness and resolution of the corneal edema [18]. Five years after the surgery, normal corneal endothelial function was still present in 10 of the 11 eyes, with significant improvements in the best corrected visual acuity [19].

Regardless, the achieved capacity for expanding human CECs is still highly limited; new sources of CECs are, therefore, sought. Recently, CECs have been shown to be derived from the neural crest [20], thus contributing to the crucial developments in the directed differentiation of embryonic and induced pluripotent stem cells (iPSCs) into CECs [21,22,23,24,25]; however, these cells have disadvantages, such as an allogenic origin, and the use of embryonic stem cells and, in the case of iPSCs, retroviral insertion or the use of Sendai virus and oncogenes. iPSCs are also difficult and time-consuming to establish and culture. Nevertheless, a huge amount of knowledge about the mechanisms that drive the differentiation towards the CEC lineage has been revealed using these approaches.

We aimed to transfer this knowledge to obtain human CECs from human adipose tissue-derived mesenchymal stem cells (hADSCs), an easier to culture cell source, which is also easy to obtain through elective liposuction, providing a high starting number of cells, with no viral or genetic manipulation necessary and, importantly, a possible autologous use.

## 2. Results

### 2.1. hADSC Differentiation into NCCs

**Figure 1:**
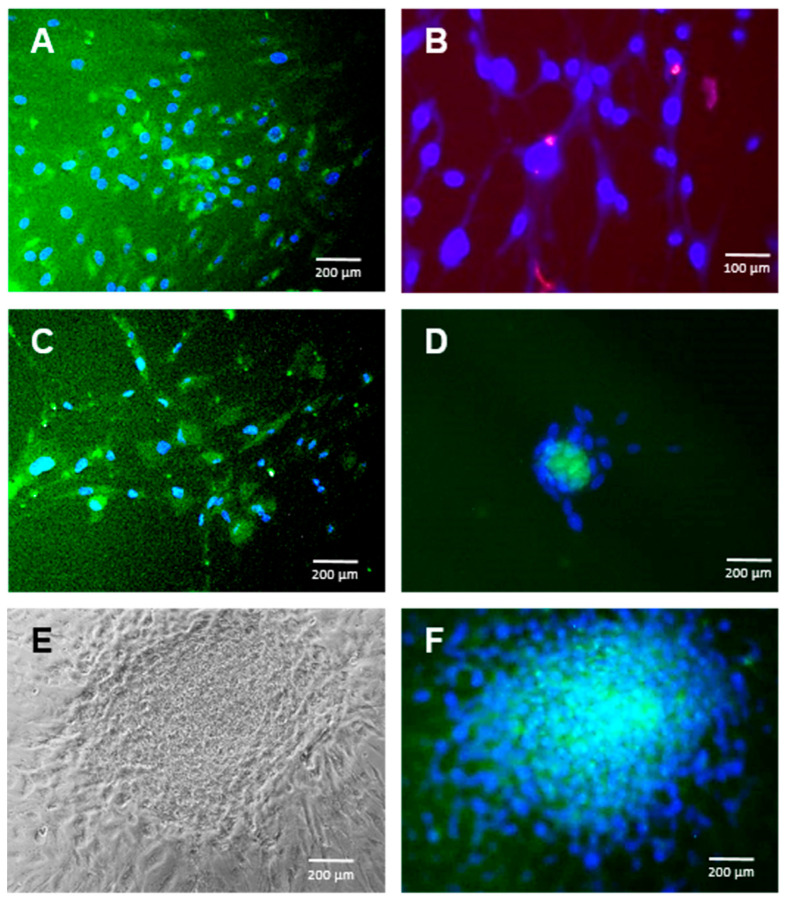
Adipose tissue-derived adult mesenchymal stem cell (ADSC) differentiation into the neural lineage. Immunofluorescence for S100 in ADSC cultures exposed to several differentiation protocols towards neural crest cells (NCC). (**A**): Jang et al. protocol at 14 days (S100 positive cells in green at the cytoplasm). (**B**): Zavan et al. protocol at 20 days (S100 positive cells in red at the Golgi apparatus). (**C**): Ali et al. protocol at 10 days (S100 positive cells in green at the cytoplasm). (**D**): Wagoner et al. protocol at 17 days (S100 positive cells in green at the cytoplasm). (**E**): Phase contrast morphology of a neurosphere generated spontaneously in Wagoner culture media at 20 days. (**F**): Same neurosphere stained for S100 (in green at the cytoplasm). Cell nuclei are stained with DAPI in blue. Bars represent 100 or 200 µm as indicated.

As expected, both previously published methods of hADSC-directed differentiation into NCCs rendered such cells in as little as 14 days (protocol of Jang et al. [26]), showing up to 57% cells positive for S100 by immunofluorescence (Figure 1A). Similar results were achieved with the Zavan [27] method in 20 days, as published, although, in this case, S100 was found only at the Golgi location, indicating an earlier differentiation stage (Figure 1B). No differences in the cellular morphology were observed.

Surprisingly, the differentiation methods previously used for iPSCs and embryonic stem cells (ESC) also rendered S100-protein-positive cells derived from hADSCs in even less time, in as little as 10 days, when using the Ali [25] conditions (Figure 1C) and in 17 days when using the Wagoner [24] conditions (Figure 1D). Culturing hADSCs in the basal medium supplemented with the Ali or Wagoner factors did not provoke S100 expression (not shown), suggesting that the embryonic stem cell medium is mostly responsible for the neuroectoderm differentiation of hADSCs. Both the Ali and Wagoner conditions generated neurospheres (Figure 1E,F).

### 2.2. hADSC Differentiation into CEC Cells

**Figure 2:**
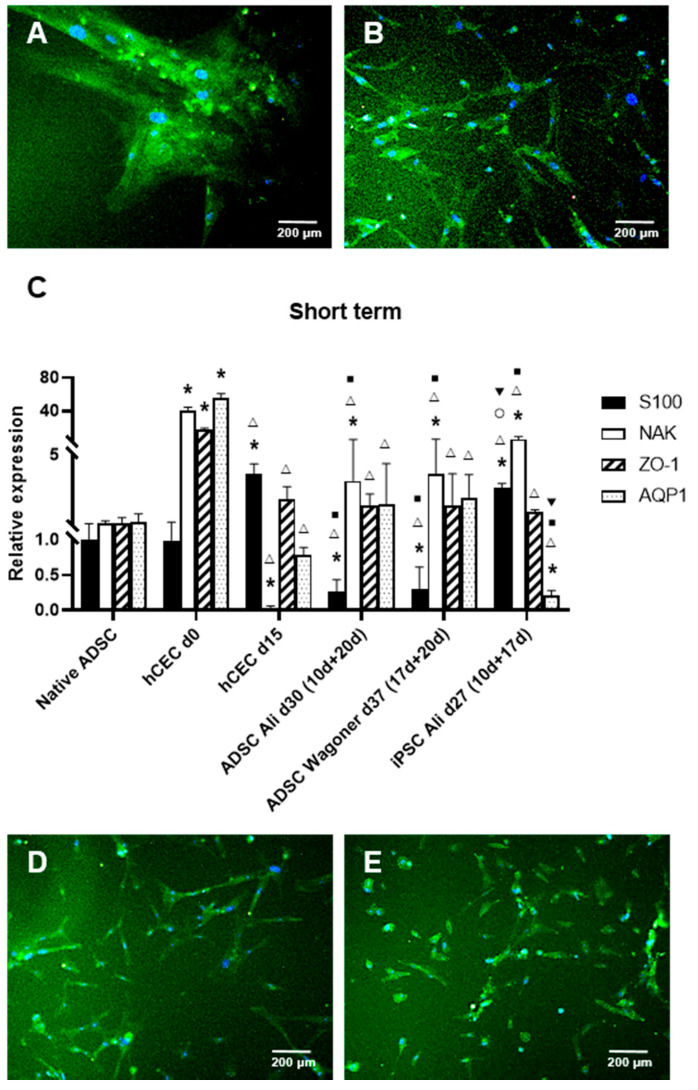
ADSC directed differentiation into corneal endothelial cells (CECs). Merge immunofluorescence images of the expression of S100 (red) and Na^+^/K^+^ ATPase (green) in ADSC cultures subjected to different protocols. Note S100 downregulation as demonstrated by lack of red fluorescence in every image (**A**): Ali protocol at 20 days of the second stage. (**B**): Wagoner protocol at 10 days of the second stage. (**C**): Quantitative RT-PCR of neural and corneal endothelial markers of ADSC-derived CEC cultures. Data are respective of the baseline expression in undifferentiated ADSCs (randomly assigned as 1). Note the significantly increased expression of Na^+^/K^+^ ATPase using both protocols and the decreased S100 expression. Comparisons can be made with human CECs directly isolated from donor corneas (d0) and cultured as usual for transplantation (d15). Moreover, compare the expression with induced pluripotent stem cells (iPSCs). (**D**): Na^+^/K^+^ ATPase-positive cells were detected at 20 days of the second stage using Zavan at first and then Ali in the second stage, also with S-100 downregulation (**E**): Na^+^/K^+^ ATPase-positive cells at 20 days of the second stage when using Jang at first and then Ali in the second stage, also with S-100 downregulation. Cell nuclei are stained with DAPI in blue. Bars represent 200 µm. * symbol indicates statistical significance difference compared to native ADSC. Δ symbol indicates statistical significance difference compared to human CECs directly isolated from donor corneas (d0). ■ symbol indicates statistical significance difference compared to human CECs cultured as usual for transplantation (d15). ○ symbol indicates statistical significance difference compared to ADSC directed differentiation into CECs with Ali protocol at 20 days of the second stage. ▼ symbol indicates statistical significance difference compared to ADSC directed differentiation into CECs with Wagoner protocol at 20 days of the second stage. (significance at *p* < 0.05).

Consequently, we proceeded to use the second stage of the Ali and Wagoner protocols in our cultures previously differentiated to NCCs as described above. Such media were able to generate Na^+^/K^+^ ATPase-positive cells in 15–20 days and 10–20 days for the second stage of the Ali et al. or Wagoner et al. protocols, respectively (Figure 2A,B). The differentiation efficiency reached 94% with the Ali protocol and 80.2% with the Wagoner protocol. Interestingly, the S100 expression was downregulated in both protocols, as demonstrated both by immunofluorescence (Figure 2A,B) and qRT-PCR (Figure 2C), whereas Na^+^/K^+^ ATPase was statistically upregulated (Figure 2C). It is noteworthy that native hADSCs also express a fair amount of other CEC markers (ZO1 and aquaporin) that maintain their levels in differentiation conditions (Figure 2C). The use of the same protocols but with iPSCs resulted in similar levels of ZO1 and aquaporin when using hADSCs with both protocols but with the upregulation of the S100 levels and aquaporin downregulation with the iPSCs (Figure 2C). A comparison with actual human CECs directly isolated from donor corneas showed that all the CEC markers, Na^+^/K^+^ ATPase, ZO1, and aquaporin, are at high levels, which decrease after 15 days of culture (non-passaged cells), showing dramatically low levels for Na^+^/K^+^ ATPase (Figure 2C). In conclusion, no statistically significant differences were encountered in the CEC markers using either the Ali or Wagoner protocols in the short term, rendering a similar CEC marker profile to that of freshly isolated CECs but at lower levels.

When using first the Zavan and then the Ali protocols in the second stage, up to 92% of the Na^+^/K^+^ ATPase-positive cells were detected at 20 days of the second stage, also with S100 downregulation (Figure 2D). When using first the Jang and then the Ali protocols in the second stage, we obtained up to 79.7% Na^+^/K^+^ ATPase-positive cells at 20 days of the second stage, also with S100 downregulation (Figure 2E).

In conclusion, all four of the protocols rendered corneal endothelium-like cells with an efficiency of at least 80%, with the Ali protocol having the higher quantitative efficiency.

### 2.3. Long-Term Culture and CEC Characterization

**Figure 3:**
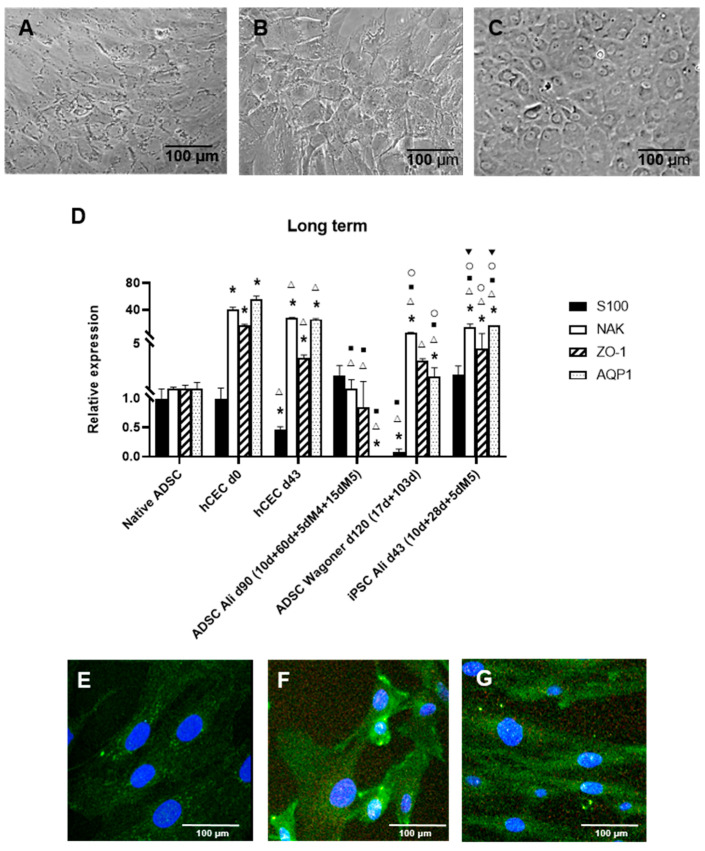
Long-term differentiation of ADSC-derived CEC characterization. (**A**): Phase-contrast im-ages showing typical CEC morphology using the Ali protocol and ADSCs. (**B**): Similar morphology using the Wagoner protocol and iPSCs. (**C**): Actual human CEC cultures at day 15. (**D**): Quantitative RT-PCR of long-term ADSC-derived CEC cultures. Data are respective of the baseline expression in the undifferentiated ADSCs (randomly assigned as 1). Note the statistically increased expression of Na^+^/K^+^ ATPase and the S100 decreased expression only with the Wagoner protocol. Comparisons can be made with human CECs directly isolated from donor corneas (d0) and cultured long term (d43). Moreover, compare the expression with iPSCs. (**E**): Undifferentiated passage 5 ADSCs with low expression of Na^+^/K^+^ ATPase protein. (**F**): Differentiated ADSCs with increased expression of Na^+^/K^+^ ATPase protein with the Ali protocol at 30 days. (**G**): Differentiated ADSCs increased the expression of Na^+^/K^+^ ATPase protein with the Wagoner protocol at 27 days. Cell nuclei are stained with DAPI in blue. Bars represent 100 µm. * symbol indicates statistical significance difference compared to native ADSC. Δ symbol indicates statistical significance difference compared to human CECs directly isolated from donor corneas (d0). ■ symbol indicates statistical significance difference compared to human CECs cultured long term (d43). ○ symbol indicates statistical significance difference compared to ADSC directed differentiation into CECs with Ali protocol at 80 days of the second stage. ▼ symbol indicates statistical significance difference compared to ADSC directed differentiation into CECs with Wagoner protocol at 103 days of the second stage. (significance at *p* < 0.05).

To obtain completely differentiated hADSC-derived CEC cultures, with a typical hexagonal and tightly packed morphology, long-term cultures were performed. Such cells were obtained with the Ali (Figure 3A) and Wagoner protocols. In our hands, the Ali protocol rendered a superior and more consistent morphology. Figure 3B shows iPSCs with the Wagoner protocol showing similar morphology, and Figure 3C shows the actual human CEC culture for comparison, showing complete hexagonal and tighter morphology.

The further long-term characterization of such cells was performed by the expression of typical CEC markers, which, interestingly, vary in actual human CECs with the culture, with high expression in directly isolated human CECs (d0, Figure 2C), decreasing in normal culturing conditions at passage 0 (d15, Figure 2C), and upregulating again after passaging at d43 (passage 1, Figure 3D). Long-term ADSC-derived CECs were positive for markers S100, Na^+^/K^+^ ATPase, and ZO1 using both protocols; however, the Wagoner protocol rendered significantly lower S100 expression than the Ali protocol, whereas the Ali protocol showed lower aquaporin expression (Figure 3D). The Wagoner expression pattern was also more similar to the iPSC-derived CECs. The comparison with actual long-term cultured CECs showed lower levels of all the markers (Figure 3D).

Interestingly, the CEC markers did not increase with the culturing time, either in the hADSC-derived CECs or the iPSC-derived CECs (compare Figure 2C and Figure 3D).

At the protein level, long-term differentiated hADSCs upregulated Na^+^/K^+^ ATPase, as assessed by immunofluorescence (compare Figure 3E with Figure 3F,G).

## 3. Discussion

In our hands, all four of the protocols for inducing the neural crest differentiation from hADSCs were successful despite the numerous differences in the culture media’s growth factor composition.

As for actual CEC differentiation, all the combinations rendered high numbers of Na^+^/K^+^ ATPase-positive cells (at least 80% with each combination or protocol used). In our hands, the Ali protocol was superior, with higher quantitative efficiency (94% Na^+^/K^+^ ATPase-positive cells).

Interestingly, typical CEC marker expression varies in actual human CECs with the culture, with high expression in directly isolated human CECs (d0, Figure 2C), decreasing in normal culturing conditions at passage 0 (d15, Figure 2C), and upregulating again after passaging at d43 at passage 1 (Figure 3D). This finding could help in the clinical use of human CECs, suggesting that using longer-term cultured cells would result in greater success.

Using the same protocols but with iPSCs, there was stronger S100 downregulation and greater aquaporin upregulation in the short term. The actual importance of these two genes in the functional performance of extraocular-cell-derived CECs remains unclear at this time, and its importance compared with the time and cost savings of using hADSCs versus iPSCs needs to be elucidated. Even with iPSCs, however, we did not completely achieve a hexagonal shape and tightly packed cells compared with actual CECs.

The time costs of the Ali and Wagoner protocols are similar, with a minimum of 25 and 27 days for achieving CEC differentiation, respectively. The financial costs of the Ali and Wagoner protocols favor the former for being less expensive due to fewer factors used in the medium (see below).

It is interesting to compare the signal transduction pathway manipulation in the protocols used for human cells [28]. FGF2 was used to promote neural induction in all the protocols. FGF2 also induces the production of collagen type I and fibronectin [29]. The β-mercaptoethanol used in the Ali and Wagoner protocols is also beneficial for the shape of CECs [30]. Similar results were obtained using all-transretinoic acid exposure of mouse ESC and human iPSCs [31].

The Wagoner and Ali protocols (as well as the McCabe et al. protocol, not compared in our study) have in common SB431542, which inhibits Lefty/activin/transforming growth factor-β pathways, inducing a loss of pluripotency associated with blocking the formation of mesodermal lineage and engaging with GSK3-mediated signaling to enhance neural induction [32]. This factor is also an important component of the McCabe et al. protocol [22].

As differential factors, the Wagoner protocol uses CHIR99021, an inhibitor of GSK-3 activity, which leads to a nuclear accumulation of β-catenin, which, in turn, activates the Wnt canonical signaling pathway, preventing terminal cell differentiation [33]. Recent studies have achieved long-term mouse CEC differentiation from mouse embryonic fibroblasts using a similar protocol with TGF-β and GSK3 inhibitors for NCC induction and Wnt inhibitors to further induce CEC differentiation [34], showing polygonal cells that proved functional in vivo.

The Ali protocol uses Noggin, a Smad inhibitor, which has a key role in suppressing endogenous BMP signals. This factor also produces a neuroepithelial cell population and induces the expression of the early eye transcription factor gene PAX6 [35]. Zhao et al. [23] used a dual Smad and Wnt inhibition first and then Wnt activation from iPSCs. In the protocol by Chen et al. [31], the exposure of mouse ESC and iPSCs to lens epithelial cell-conditioned medium exerted endothelial differentiation, but there is obviously no available composition data.

The comparison of the Ali and Wagoner protocols revealed that the latter has more components in the basal medium than the former, components such as ascorbic acid, transferrin, heregulin β-1, and IGF-1. Ascorbic acid is an important co-factor for collagen synthesis. However, concentrations >55 µg/mL have a detrimental effect on cell growth [36]. To help this deficiency in the cell growth caused by ascorbic acid, Wagoner et al. used human transferrin given that the 2% FBS was insufficient and there is a low affinity of bovine transferrin for the human transferrin receptor [37].

In conclusion, we have effectively presented several protocols capable of the directed differentiation of hADSCs to human CECs. In our hands, the Ali protocol was the best adapted to such differentiation in terms of efficiency, time, and financial cost; however, the Wagoner protocol was superior for CEC marker expression. Our results broaden the type of cells of autologous extraocular origin that could be employed in the clinical setting for corneal endothelial deficiency.

## 4. Materials and Methods

### 4.1. Isolation of hADSCs

After granting their written informed consent, lipoaspirate from three female donor patients (ages 41, 44, and 47 years) who underwent elective liposuction (body mass index 28.1, 28.3, and 28.4) was obtained by a plastic surgeon. The patients were otherwise healthy and were not undergoing drug therapy, except for one patient who took fluoxetine for depression. The isolation protocols were approved by the Institutional Review Board of La Paz Hospital (Madrid, Spain) and were in accordance with the Declaration of Helsinki (2000) of the World Medical Association.

Active infection by HIV, hepatitis C virus, and syphilis was ruled out by serological analyses. The isolation was performed previous to the COVID-19 pandemic; no test for such infection was, therefore, performed. The hADSCs were isolated as previously described [38] and stored in the biobank of La Paz Hospital. A previous study by our group demonstrated that this protocol was effective in isolating hADSCs capable of multipotent lineage differentiation [38]. Briefly, the adipose tissue from the human liposuction was washed with phosphate buffered saline (PBS) and digested with 0.09% collagenase I in PBS (Gibco-BRL, Grand Island, NY, USA) for 45 min at 37 °C under gentle stirring. The solution was then inhibited with fetal bovine serum (FBS; Gibco) and centrifuged at 300× *g* for 10 min to obtain the adipose-tissue stromal vascular fraction of the pelleted cells and to discard the floating adipocytes. The pellets were treated with erythrocyte lysis buffer (160 mM NH4Cl; 10 mM KHCO3; 1 mM EDTA; all from Sigma-Aldrich, St. Louis, MO, USA) for 15 min at RT. The pellets were washed with PBS and seeded at 1 × 10^6^ cells per plate into 10-cm plates (Corning) containing standard media consisting of Dulbecco’s Modified Eagle Medium (DMEM; Gibco), supplemented with 10% FBS, Na-pyr 110 mg/L (Gibco), Glutamax 862 mg/L (Gibco), and 1% penicillin-streptomycin (Sigma). The hADSCs used in the present study were from passages 1 to 5.

### 4.2. Differentiation Protocols

**Figure 4:**
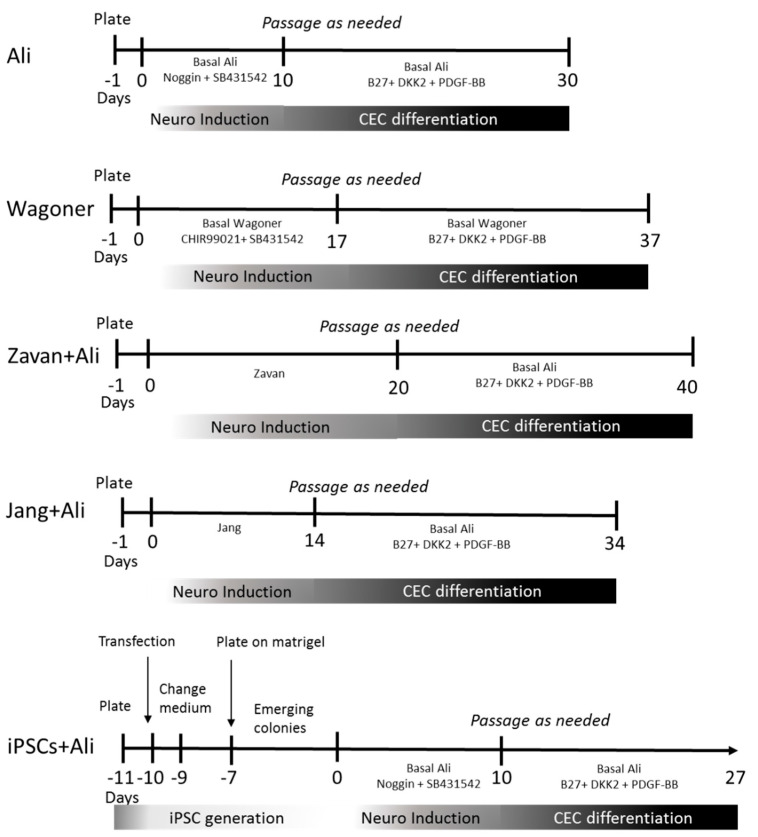
Timeline of the protocols used for ADSC and iPSC differentiation towards CEC. Numbers indicate minimum days in culture, minus signed numbers indicate days in culture previous to differentiation protocol.

We used hADSCs to generate CECs by modifying previously published procedures. We employed two protocols that demonstrated the induction of neural differentiation from hADSCs and two other protocols that induced CEC differentiation from iPSCs with a previous neural differentiation step. For a timeline of all protocols employed, see Figure 4.

To induce neural differentiation, we used the protocols described by Jang et al. [26] and the protocol described by Zavan et al. [27] for ADSC differentiation to neural crest cells (NCC), with slight differences: The hADSCs were grown following the Jang et al. protocol using DMEM (Thermo Fisher Scientific, Waltham, MA, USA) containing 1% FBS (GE Healthcare, Chicago, IL, USA), 1% penicillin/streptomycin (GE Healthcare), and supplemented with 100 ng/mL fibroblast growth factor (FGF2, Thermo Fisher Scientific) for seven days. The cells were then incubated in the presence of 10 μM forskolin (Merck KGaA, Darmstadt, Germany) for an additional seven days.

In addition, the hADSCs were grown following the Zavan et al. protocol using DMEM:HAM F12 (3:1) (Thermo Fisher Scientific) containing 10% FBS, 1% penicillin/streptomycin, and supplemented with 20 ng/µL epidermal growth factor (EGF, Thermo Fisher Scientific) and 40 ng/µL FGF for up to 20 days.

To induce CEC differentiation, we used the protocols described by Ali et al. [25] and the protocol described by Wagoner et al. [24] for iPSC differentiation into CECs, with slight differences: the Ali et al. protocol makes use of the dual Smad inhibitor medium, containing 500 ng/mL human recombinant Noggin (R&D Systems, Minneapolis, MN, USA) and 10 uM SB431542 (Merck KGaA) in a basal medium of 80% DMEM:HAM F12 (1:1), 20% knockout serum replacement (KSR; Life Technologies, Carlsbad, CA, USA), 1% non-essential amino acids (Thermo Fisher Scientific), 1 mM L-glutamine (StemCell Technologies, Inc., Vancouver, BC, Canada), 0.1 mM β-mercaptoethanol (Merck KgaA), and 8 ng/mL of FGF2. Cultures were maintained in this medium for 2 to 10 days, which was then replaced by corneal endothelial medium containing 0.1X B27 supplement (Thermo Fisher Scientific), 10 ng/mL recombinant human platelet-derived growth factor-BB (PDGF-BB; PeproTech, Rocky Hill, NJ, USA), and 10 ng/mL recombinant human Dickkopf-related protein 2 (DKK-2, R&D Systems) in the basal medium for 15 to 20 days.

The Wagoner et al. protocol makes use of neural crest medium containing induction factors: 10 µM SB431542 and 3 µM CHIR99021 (Miltenyi Biotec Inc.; Bergisch Gladbach, Germany) in a basal medium of DMEM:HAM F12 (1:1), 2% FBS, 2 mM GlutaMax (Thermo Fisher Scientific), 0.1 mM MEM non-essential amino acid solution, 1x trace elements A, B, and C (Thermo Fisher Scientific), 0.1 mM β-mercaptoethanol, 50 µg/mL Na^+^-l-ascorbate (Merck KGaA), 10 µg/ mL transferrin (Merck KGaA), 10 ng/mL recombinant human Heregulin β-1 (PeproTech), 200 ng/mL recombinant human LONGR3 IGF-I (Merck KGaA), 8 ng/mL recombinant human FGF2, and 1% penicillin/streptomycin. We cultivated hADSCs in this medium for 3 to 17 days. The medium was then switched to corneal endothelial induction medium containing the basal medium and the same corneal factors as the previously mentioned Ali et al. protocol for 10 to 30 days.

The Ali et al. and Wagoner et al. protocols use basal medium for iPSCs; we, therefore, used either this previously described basal medium or hADSC basal medium, i.e., DMEM containing 10% FBS, GlutaMax (862 mg/L), Na^+^-pyruvate (110 mg/L) (Gibco), and 1% penicillin/streptomycin.

After the neural differentiation with the Jang et al. or Zavan et al. protocols, we induced corneal differentiation using the second stage of the Ali et al. or Wagoner et al. corneal endothelial induction media protocols.

### 4.3. Quantitative Real Time Reverse Transcriptase-Polymerase Chain Reaction (qRT-PCR)

Total RNA was isolated from cell cultures using TRIzol reagent (Merck, Darmstadt, Germany), and 1 µg of the total RNA was used for cDNA synthesis with SuperscriptTM II Reverse Transcriptase Kit and Random hexamers primer (Invitrogen, Carlsbad, CA, USA) according to the manufacturer’s instructions. Quantitative polymerase chain reactions were set up in duplicate by using PowerUp SYBR Green Master Mix (Applied Biosystems, Foster City, CA, USA) and analyzed in a CFX96 TouchTM real-time PCR equipment (Bio-Rad, Hercules, CA, USA). The conditions for qPCR were 2 min at 50 °C for the uracil-DNA glycosylase activation, 2 min at 95 °C for the polymerase activation, followed by 40 cycles of amplification with 15 s at 95 °C, 15 s at 60 °C, and 1 min at 72 °C. Each gene’s expression was normalized using β-actin as the endogenous control. The respective gene expression change of each gene was calculated relative to the hADSCs in the fifth untreated passage as control samples using the comparative threshold cycle (Ct) method with the formula 2^-ΔΔCt^.

The real-time PCR primer sequences were as follows: S100 forward, 5ʹ-GGC GAA TGTGACTTCCAGGAA TTCAT-3′, and reverse 5′-AGGGTGCCCCGGGGTAATTTCTGTAG-3′ [39]; Na^+^/K^+^ ATPase forward, 5′-GGTCCCAACGCCCTCACTC-3, and reverse 5′-ACCACACCCAGGTACAGATTATCG-3′ [23]; ZO1 forward, 5′-CCCCACTCTGAAAATGAGGA-3′, and reverse 5ʹ-GGGAACAACATACAGTGACGC-3′ [40]; Aquaporin1 forward 5′- ACCTCCTGGCTATTGACTACA-3′, and reverse 5′- CCCTTCTATTTGGGCTTCATCT-3′ [24]; β-actin forward, 5′-GTGACGAGGCCCAGAGCAAGAG-3′, and reverse 5′-ACGCAGCTCATTGTAGAAGGTGTGG-3′ [41].

For comparison, human CECs were obtained from three corneal donors. The CECs were unsuitable for transplantation and were obtained from the eye bank of the Lions Eye Institute (Tampa, FL, USA). The human tissue used in the study had prior consent for use in research, and the Helsinki Declaration for biomedical research involving humans (2000) was adhered to throughout the study. None of the donors were from a vulnerable population, and all donors or next of kin provided written informed consent that was freely given. The isolation protocols were approved by the Institutional Review Board of Ramon y Cajal Hospital (Madrid, Spain). Active infection by HIV, hepatitis C virus, and syphilis was ruled out by serological analyses. The isolation was performed previous to the COVID-19 pandemic; no test for such infection was, therefore, performed. CECs were isolated and used uncultured (d0), short-term cultured in M4 and M5 media (15 days), as previously published by Peh et al. [12] and Arnalich-Montiel et al. [16], and passaged and cultured longer term (43 days). Briefly, human CECs were plated at 3 × 10^3^ cells/mm^2^ in fibronectin-coated plates in M4 medium consisting of 50% Ham’s F12 media, 50% M199, 5% FBS, 2 µg/mL ascorbic acid, 10 ug/mL insulin, 5.5 ug/mL transferrin, 5 ng/mL selenium, 1% penicillin/streptomycin (P/S), and 250 ng/mL amphotericin B until reaching confluence. Cultures were then maintained in M5 medium consisting of Human Endothelium Serum Free Medium (Gibco), 5% FBS, 1% P/S, and 250 ng/mL amphotericin B. RNA isolation and qRT-PCR were performed in the same manner as for differentiated hADSCs.

In addition, human iPSCs were generated from hADSCs by transfection. Briefly, hADSCs were seeded 24 h before transfection at a density of 5 × 10^4^ cells/cm^2^ in 6-well plates with basal medium (DMEM, GlutaMax, 10% FBS, 1% P/S). Plasmids encoding the transgenes Oct4, Sox2, Klf4, and cMyc (Addgene) were introduced using TurboFect™ reagent (Thermo Fisher) following the manufacturer’s instructions. Briefly, 11 µg of plasmid DNA (6 µg of MOS and 5 µg of MMK from Addgene) was diluted in 400 µL of serum-free DMEM, mixed with 6 µL of transfection reagent, and incubated for 20 min at RT. The mixture was added drop-wise to each well and incubated at 37 °C for 24 h. The cells were maintained in basal medium for an additional 24 h, which was gradually replaced by ReproTeSR™ medium (Stem Cell Technologies) for the next two days. On day 3, the surviving cells were reseeded on Matrigel coated plates, and the medium was changed daily until colonies emerged approximately at 10 days post-transfection. At that time, the medium was changed to NCC differentiation medium and later to CEC differentiation medium as with the non-induced hADSCs (Figure 4).

### 4.4. Immunocytochemistry

To show neuroectodermal differentiation, we used S100, which is known to have a wide distribution in human tissues, including glia, neurons, Schwann cells, and notochords among others. To show CEC differentiation, we checked for Na^+^/K^+^ ATPase protein expression as part of an essential component of the endothelial corneal “pump-leak” mechanism, enriched in CECs.

The cells were fixed with ice cold 100% ethanol at RT for 5 min. Blocking was achieved with 5% goat serum and 1% bovine serum albumin in PBS for 30 min. Cells were incubated with 1:50 S100 (Dako Omnis) rabbit antibody and 1:400 mouse Na^+^/K^+^ ATPase antibody (Santa Cruz Biotechnology, Dallas, TX, USA) overnight at 4 °C. The labelled cells were rinsed the next day in PBS and subsequently incubated in 1:200 goat anti-rabbit IgG Texas Red (Vector Laboratories) or 1:500 goat anti-mouse IgG antibody biotinylated-avidin FITC (Vector Laboratories, Inc., Burlingame, CA, USA) secondary antibodies, respectively, for 1 h at RT. The nuclei were counterstained with 4′,6-diamidine-2′-phenylindole dihydrochloride (DAPI; Merck KGaA). Images were captured using an Axiovert 200 fluorescence Zeiss microscope or a confocal SP5 microscope (Leica Microsystems). Manual counting was used to assess % of immunofluorescence positive cells by counting 15 random confocal fields at 630x magnification.

### 4.5. Statistical Analysis

qRT-PCR data were compared with Student’s *t*-tests using GraphPad Prism 8.0 (GraphPad Software, La Jolla, CA, USA). Data are reported as mean ± standard deviation of individual experiments including at least three replicates. The *p* value for statistical significance in this evaluation was set to 0.05.
